# Spatial-Temporal Signals and Clinical Indices in Electrocardiographic Imaging (I): Preprocessing and Bipolar Potentials

**DOI:** 10.3390/s20113131

**Published:** 2020-06-01

**Authors:** Raúl Caulier-Cisterna, Margarita Sanromán-Junquera, Sergio Muñoz-Romero, Manuel Blanco-Velasco, Rebeca Goya-Esteban, Arcadi García-Alberola, José Luis Rojo-Álvarez

**Affiliations:** 1 Department of Signal Theory and Communications, Telematics and Computing Systems, Rey Juan Carlos University, Fuenlabrada, 28943 Madrid, Spain; raul.caulier@urjc.es (R.C.-C.); margarita.sanroman@urjc.es (M.S.-J.); sergio.munoz@urjc.es (S.M.-R.); rebeca.goyaesteban@urjc.es (R.G.-E.); 2 Center for Computational Simulation, Universidad Politécnica de Madrid, Boadilla, 28223 Madrid, Spain; 3 Department of Signal Theory and Communications, Universidad de Alcalá, Alcalá de Henares, 28805 Madrid, Spain; manuel.blanco@uah.es; 4 Arrhythmia Unit, Hospital Clínico Universitario Virgen de la Arrixaca de Murcia, El Palmar, 30120 Murcia, Spain; arcadi@secardiologia.es

**Keywords:** electrocardiographic imaging, signal processing, cardiac indices, spatial-temporal processing, infarction, fragmentation

## Abstract

During the last years, Electrocardiographic Imaging (ECGI) has emerged as a powerful and promising clinical tool to support cardiologists. Starting from a plurality of potential measurements on the torso, ECGI yields a noninvasive estimation of their causing potentials on the epicardium. This unprecedented amount of measured cardiac signals needs to be conditioned and adapted to current knowledge and methods in cardiac electrophysiology in order to maximize its support to the clinical practice. In this setting, many cardiac indices are defined in terms of the so-called bipolar electrograms, which correspond with differential potentials between two spatially close potential measurements. Our aim was to contribute to the usefulness of ECGI recordings in the current knowledge and methods of cardiac electrophysiology. For this purpose, we first analyzed the basic stages of conventional cardiac signal processing and scrutinized the implications of the spatial-temporal nature of signals in ECGI scenarios. Specifically, the stages of baseline wander removal, low-pass filtering, and beat segmentation and synchronization were considered. We also aimed to establish a mathematical operator to provide suitable bipolar electrograms from the ECGI-estimated epicardium potentials. Results were obtained on data from an infarction patient and from a healthy subject. First, the low-frequency and high-frequency noises are shown to be non-independently distributed in the ECGI-estimated recordings due to their spatial dimension. Second, bipolar electrograms are better estimated when using the criterion of the maximum-amplitude difference between spatial neighbors, but also a temporal delay in discrete time of about 40 samples has to be included to obtain the usual morphology in clinical bipolar electrograms from catheters. We conclude that spatial-temporal digital signal processing and bipolar electrograms can pave the way towards the usefulness of ECGI recordings in the cardiological clinical practice. The companion paper is devoted to analyzing clinical indices obtained from ECGI epicardial electrograms measuring waveform variability and repolarization tissue properties.

## 1. Introduction

Despite technological advances and interpretation of arrhythmias using the skin-surface recorded Electrocardiogram (ECG), the intracardiac Electrograms (EGM) are still today the most reliable method to identify the arrhythmia mechanisms in the Electrophysiological Studies (EPS). EGM signals represent cardiac potentials recorded from electrodes in direct contact with the cardiac tissue, and they register the bioelectric activation of specific locations within the cardiac chambers. This provides electrophysiologists with an accurate instrument to localize and characterize the arrhythmias, which can overcome many of the limitations of the surface ECG in the evaluation of arrhythmias [1,2]. The registration of a set of EGMs begins by obtaining the voltage difference between two different electrodes of a catheter systems: from a set located inside the heart and from another indifferent electrode used as ground. The recording of a bipolar EGM is performed as the difference between two close electrodes, while for unipolar EGMs, either a far electrode or the average of the surface electrodes can be taken as reference. Subsequently, this difference is amplified and filtered to remove artifacts, such as high-frequency noise, breathing, or heart movements, using high-pass filters, low-pass filters, and other well-known signal processing methods. Finally, this amplified and filtered potential difference is graphically represented for the interpretation of the electrophysiologist during the EPS [3,4].

Modern medicine relies heavily on noninvasive imaging modalities to guide therapy, such as computed tomography (CT) or magnetic resonance imaging (MRI) as anatomical image modalities or ultrasounds (echocardiogram) as cardiac functioning image modality. An equivalent modality for imaging cardiac arrhythmias in terms of bioelectric activity was not fully available until the Electrocardiographic Imaging (ECGI) arrival. Recent studies on ECGI have led to the development of noninvasive techniques, which allows to reconstruct the potentials of the heart surface from many simultaneous measurements of potentials on the body surface [5,6,7,8,9,10,11,12]. Whereas traditional noninvasive ECG techniques have limited ability to determine the location of electrical events in the heart with acceptable resolution, the ECGI has shown the ability to improve the noninvasive reconstruction of epicardial EGM and isochronic potentials during EPS [13,14,15,16]. In ECGI, a multi-electrode vest is first used to record some few hundreds of simultaneous signals of body-surface potentials. Then, these torso potentials are used with geometrical information from a CT or MRI scan in order to reconstruct the electrical potentials from mathematical algorithms, in which these potentials closely correspond to unipolar EGMs, and from them, activation sequences and repolarization patterns on the epicardium or endocardium of the heart can be estimated.

The scope and limitations of current ECGI systems have been recently scrutinized in terms of comparisons with maps and clinical parameters provided by current intracardiac navigation systems [17], concluding that ECGI algorithms and subsequent signal processing techniques need to be improved to make the resulting maps clinically relevant. Therefore, further work is necessary both in signal supervision and processing and in the methods used to estimate the parameters. Other recent works have pointed to the dependence of clinical validation studies in different groups and commercial systems on the technical implementation and ECGI algorithm choices, such as boundary element method (BEM) and the finite element method (FEM) [18], and on validation methods, such as technical, pathological, or clinical validation [19]. Our aim is to show that the conventional analysis of ECGI-estimated potentials based on separate signal processing for each signal followed by map representation of some individually measured parameter has to be revisited in order to account for the unprecedented spatial richness of simultaneous signals. This emphasis on the spatial-temporal digital signal processing should be scrutinized for the conventional cardiac signal stages. In this setting, an issue that deserves special attention is the criteria for obtaining bipolar configurations for ECGI-derived EGM, which is not a trivial problem. So far, the ECGI-derived EGMs were computed using the virtual bipolar or Laplacian bipolar, which consider an infinitesimal distance between electrodes [20,21]. However, it is needed to establish a new ECGI-derived EGM in order to increase the scope and usefulness in the clinical practice of cardiac arrhythmia detection and treatment, where the bipolar EGMs are computed with the difference between the unipolar EGMs registered with two electrodes located at a certain distance.

For these purposes, we started by analyzing in this paper the basic stages of conventional cardiac digital signal processing and scrutinized the spatial-temporal implications in ECGI scenarios. Specifically, the stages of baseline wander removal, low-pass filtering, and beat segmentation and synchronization were considered. Note that the analysis was done simultaneously for all the signals, given that the epicardial potentials were obtained simultaneously from the surface electrodes. We then aimed to identify a mathematical operator, thte so-called Digital Signal Processing Operator (DSPO), in order to provide suitable bipolar EGM signals from the ECGI-estimated potentials on the epicardium. Experiments were run with detail in two cases: one from an infarction patient and another from a control subject. Several instruments were used to analyze the spatial-temporal consistency of these basic properties. After scrutinizing these preprocessing and acquisition conditions, the companion paper [22] is devoted to giving further and detailed information on clinical indices on EGM morphology, depolarization, and repolarization properties in terms of their spatial organization and spatial-temporal consistency as well as on the effect of bipolar configurations on them.

The structure of the paper is as follows. First, the usual signal processing stages for the ECGI systems are presented, where the notation used in the article is detailed. Subsequently, the following concepts are described: data used; experiments performed on their ECGI-derived signals; and the results obtained for each experiment, including a separate section for (a) spatial-temporal preprocessing properties, (b) spatial consistency of ECGI-estimated bipolar EGM, and (c) need of time delay for ECGI-estimated bipolar EGMs.

## 2. Methods and Materials

In this section we introduce the notation for the potentials observed when measuring surfaces and the processing operations performed on them. We after describe several of the possible DSPOs that can be used to obtain the bipolar potentials for their subsequent testing in the experimental section. We also present a methodology for a time-delay analysis. Finally, we describe the data sets used in this work.

### 2.1. Notation and ECGI Signal Preprocessing Stages

The notation used in this work can be described as follows. Let *S* denote a two-dimensional continuous surface (or manifold) in a three-dimensional space, and let ${\bar{r}}^{S}$ be the set of points in this surface, which is defined as

(1) $${\bar{r}}^{S}\equiv \left\{\bar{r}\in S,\;\;S\in R^{3}\right\}$$

where $\bar{r}$ stands for the position vector of any point in the three-dimensional space. In our case, surface *S* can be either the epicardium (denoted as *E*) or the torso (denoted as *T*). In terms of the electrical potential changes in space and time, denoted as $v({\bar{r}}^{s},t)$, we denote potential fields on the epicardium and on the torso as

(2) $$v_{E}\equiv \left\{v\left({\bar{r}}^{E},t\right),\;{\bar{r}}^{E}\in E\right\}\;\mathrm{and}\;v_{T}\equiv \left\{v\left({\bar{r}}^{T},t\right),\;{\bar{r}}^{T}\in T\right\}$$

The data model used in the study corresponds to a conventional cardiac signal model in which the potentials measured on the torso or on the epicardium will be the actual potentials of the bioelectric field plus some noise with different origins; therefore, the measured potential can be denoted as

(3) $$v_{m}\left({\bar{r}}^{S},t\right)=v\left({\bar{r}}^{S},t\right)+N\left({\bar{r}}^{S},t\right)+B\left({\bar{r}}^{S},t\right)+R\left({\bar{r}}^{S},t\right)$$

where N,B, and *R* represent the additive Gaussian noise, the baseline noise, and other noise contributions, respectively. To reduce these types of noise, different operators are usually defined, here denoted as $\Phi_{N}$ and $\Phi_{B}$ for high-frequency noise and baseline removal, respectively; therefore,

(4) $$\hat{v}\left({\bar{r}}^{S},t\right)=\Phi_{B}\left(\Phi_{N}\left(v_{m}\left({\bar{r}}^{S},t\right)\right)\right),$$

where $\hat{v}$ denotes estimated potentials. For better visualization of some of the results, the M-mode representation of the EGM is used here [23], which is defined as the time–space representation of a discrete set of signals measured in a line of points $l_{S}$ on the surface under analysis. Accordingly, we can study the spatial-temporal evolution on path $l_{S}$ through the M-mode representation of the corresponding time signals, which is given by

(5) $$M_{v}\left(d,t\right)=v\left(l_{S},t\right),\;l_{S}\in {\bar{r}}^{S}\left(d_{start},d_{end}\right)$$

where *d* is the Euclidean distance between consecutive points in the spatial line $l_{S}$. This representation has been used to scrutinize sets of time signals that are related through a second dimension, often the spatial dimension, so that spatial-temporal structures can be readily quantified and visualized, as it will be seen in the experiments.

A surface is represented as a geometrical mesh, which is the result of discretizing surface *S* as a consequence of measuring the coordinates of the potential measurement point in the space, and then the notation changes as follows. A single point *i* on the mesh surface is denoted as

(6) $${\bar{s}}_{i}={\bar{r}}^{S}\cdot \delta \left(\bar{r}-{\bar{r}}_{i}\right)={\bar{r}}^{S}\cdot \delta \left(x-x_{i},y-y_{i},z-z_{i}\right)$$

where $\delta ({\bar{r}}_{i})$ is a Dirac’s delta function in the spacial domain and $\left\{{\bar{s}}_{i},i=1,\ldots ,N\right\}$ is a discrete set of points belonging to surface *S*. Accordingly, the mesh is given by the set of points measured in *S*, i.e.,

(7) $${\bar{r}}_{m}^{S}={\bar{r}}^{S}\sum_{i=1}^{M}\delta \left(\bar{r}-{\bar{r}}_{i}\right)$$

where *M* is the number of available points in the mesh.

The extracellular potential in quasielectrostatic conditions is a spatial-temporal function and is denoted here as $v(\bar{r},t)$, where $\bar{r}$ is the spatial position and *t* is the time. Therefore, the potential measured on the epicardium can be denoted as $v_{m}\left({\bar{r}}^{E},t\right)$. The set of potentials measured in the epicardium mesh at time instant *t* is $\left\{v_{m}\left({\bar{s}}_{i},t\right),i=1,\ldots ,M\right\}$, which corresponds to the unipolar EGM that is ideally measured with a single electrode of the catheter at point ${\bar{s}}_{i}$. A similar notation can be established in terms of delta functions for the potential field measured or estimated in different volumes or surfaces of interest.

### 2.2. DSPO for Bipolar Potentials

The potential difference recorded between two different electrodes of a catheter represents the electrical activation of the myocardium. The two electrodes are usually set in different positions of the electrophysiological catheter: The first one is a distal electrode, located at the tip of the catheter, and it is known as the exploratory electrode (positive pole). The second one, referred to as the reference electrode, is located at a close position from the distal electrode and is defined to be the negative pole. The difference between the exploration and the reference electrodes generates the bipolar EGM signals.

**Figure 1:**
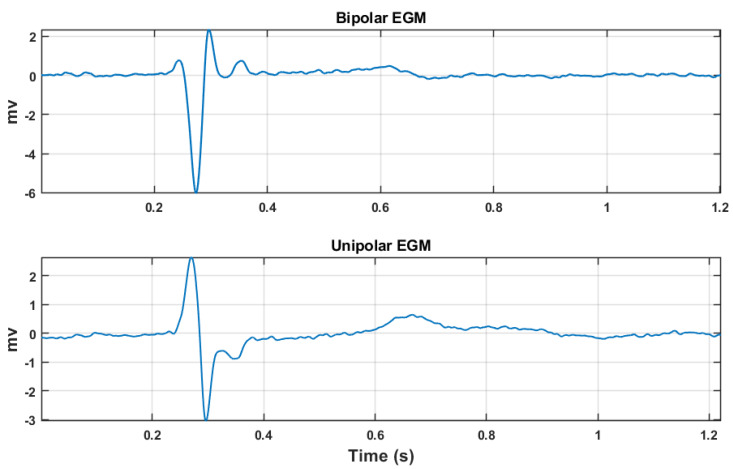
This figure depicts two Electrogram (EGM) signals taken from Electrophysiological Studies (EPS) at Hospital Virgen de la Arrixaca de Murcia in Spain. They are shown as examples of actual EGMs, though they correspond to a patient not related with the current study. The upper graph was recorded with the bipolar EGM configuration, and the lower one was recorded with the unipolar EGM configuration.

There are two types of EGM signals, which are determined depending on the distance between the two electrodes (see Figure 1). First, unipolar EGMs are obtained when the reference electrode is located at a theoretically infinite distance or far away enough to be considered as indifferent or mass. This type of signal is high-pass filtered at a cutoff frequency in the range of 0.005–0.5 Hz to eliminate baseline wander and is low-pass filtered at 500 Hz to remove out-of-band noise. Furthermore, it is also possible to filter out these unipolar EGMs between 30 to 100 Hz to detect signals of low amplitude in scar regions or in regions with slow conduction in some arrhythmias [24]. These EGMs are normally used in EPS to identify the local activation time, i.e., the moment when the depolarization front activates the myocardium located just below the explorer electrode, though it may not be carried out through visual inspection as it requires some additional computations, which are normally performed for this purpose in intracardiac navigators. An important characteristic of these unipolar EGMs is that they register a combination of local and global electrical activities with a contribution of distant electrical activity (far field) that decreases with the square of the distance to the exploration electrode [25]. Second, bipolar EGMs are obtained when both electrodes are close to each other, so that the potential difference recorded by the signal registers the local myocardial electrical activity. In bipolar EGM, the distance between the two electrodes is directly related to the section of the cardiac tissue in which the potential difference of the myocardial tissue is established. The local activity is more accurately recorded because the presence of a far field is reduced, given that the far field captured by both electrodes is similar and that it will be canceled by the difference. In most EPS, the bipolar EGMs are obtained with catheters with interelectrode distances of 1 cm or less, usually 5 mm, and even 1 mm in mapping and cartography procedures [26]. Bipolar EGMs are high-pass filtered with a cutoff frequency in the range of 10–40 Hz (normally 30 Hz) and are low-pass filtered using a cutoff frequency of 250–500 Hz [24], and they are used, for example, in reentry arrhythmias in order to measure the activation sequence even from small-amplitude and fractionated signals in low conduction areas.

**Figure 2:**
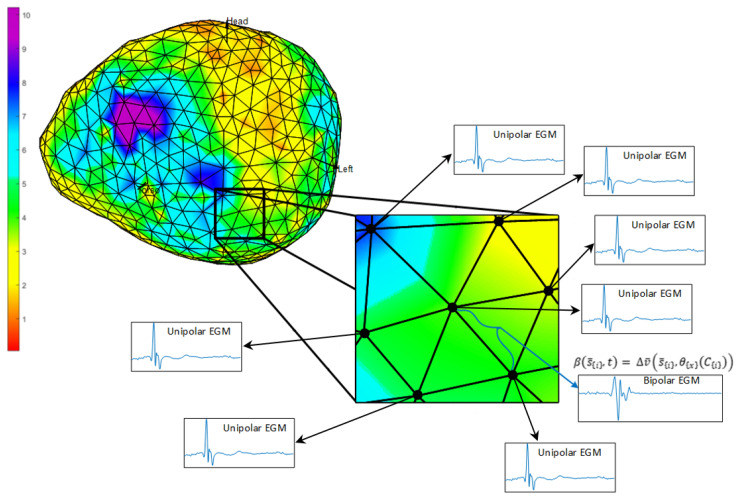
Unipolar EGM maximum potential map (color bar in mV) representing the ambiguity for the calculation of bipolar EGMs associated to the central point in terms of its nearest neighbors.

As previously pointed out, bipolar EGMs are obtained in EPS with two close electrodes located in the same catheter. Conversely, in ECGI studies, we have a mesh of points that covers the cardiac tissue, where the recorded potential at each point can be seen as a unipolar EGM. In this work, we consider the closer neighbor points to a given one as possible candidates to calculate the bipolar EGM associated to that central point, as depicted in Figure 2. The definition of the bipolar EGM associated to a given location conveys several practical problems: First, there is an ambiguity due to the diversity of the closer neighboring points. Second, the distance between the closer neighboring points and the reference point of the ECGI mesh can be larger than those of two neighbor electrodes in a conventional catheter used in clinics. Finally, the direction of the impulse propagation over the endocardial mesh can have an impact depending on the orientation of the two recording potentials, hence affecting the amplitude properties of the calculated bipolar EGM.

Let us define, in a surface mesh *S*, the set of indices $\left\{\eta_{i}\right\}$ corresponding to the nodes or points connected to a given node or point ${\bar{s}}_{i}$ in that surface mesh:

(8) $$\eta_{i}\equiv \left\{j\;/\;{\bar{s}}_{j}\;\mathrm{is}\;a\;\mathrm{connected}\;\mathrm{neighbor}\;\mathrm{to}\;{\bar{s}}_{i}\right\}$$

Thus, the set of neighbor points connected to ${\bar{s}}_{i}$ can be defined as

(9) $$C_{i}\equiv \left\{{\bar{s}}_{\eta_{i}}\right\}$$

That is, the set $C_{i}$ is composed of the bunch of points connected to the *i*th one in the mesh triangulation.

A bipolar EGM is denoted and calculated as

(10) $$v\Delta \left({\bar{s}}_{i},{\bar{s}}_{k},t\right)=v\left({\bar{s}}_{i},t\right)-v\left({\bar{s}}_{k},t\right)$$

where ${\bar{s}}_{i}$ and ${\bar{s}}_{k}$ are either two adjacent electrodes in the same catheter on the endocardium or epicardium surface or two points in the corresponding cardiac mesh from ECGI modality.

For our study, we want to stress the spatial bivariate dependence of Δv(·,·,t) and to study the optimal configuration in order to obtain a bipolar EGM which better fits with the expected clinical requirements in the usual practice, which should include similar morphologies for unipolar and bipolar EGMs when measured from catheters and when estimated from ECGI and similar amplitudes according to fragmentation criteria in clinical guidelines.

For this purpose, we define $v({\bar{s}}_{i},t_{w})$ as the temporal dependence of the epicardial potential at a given time window $t_{w}\equiv \left[t_{in},t_{end}\right]$:

(11) $$v\left({\bar{s}}_{i},t_{w}\right)\equiv \left\{v\left({\bar{s}}_{i},t\right),t\in t_{w}\right\}$$

where $t_{in}$ and $t_{end}$ correspond to the starting and ending time instants, respectively, of the heartbeat under study. Then, the bipolar EGM can be expressed as

(12) $$\beta \left({\bar{s}}_{i},t_{w}\right)=\Delta v\left({\bar{s}}_{i},\theta_{x}\left(C_{i}\right),t_{w}\right)=v\left({\bar{s}}_{i},t_{w}\right)-v\left(\theta_{x}\left(C_{i}\right),t_{w}\right)$$

where ${\bar{s}}_{k}=\theta_{x}\left(C_{i}\right)$ is the suitable closest neighbor point taken among those in the set $C_{i}$, which has been chosen by the DSPO $\theta_{x}$. The subscript *x* denotes the different DSPO criterion to be considered.

The set of DSPOs considered here for their analysis are the following: (a) maximum and minimum amplitude, (b) maximum and minimum distance, (c) average from the closest neighbors, and (d) random closest neighbors. Table 1 describes their mathematical definitions and notations. Given that the EGM amplitude is a key parameter in many EPS criteria, we will analyze the statistical and spatial properties of this feature for each of these processing operators.

### 2.3. Time Derivative and Time Delay Analysis

The morphology of a bipolar EGM is highly relevant since it is the object of study for electrophysiologists. The fragmentation, the deflection width, and the amplitude of bipolar EGM are electrophysiological parameters that are carefully studied in EPS. These parameters are used in EPS to determine the different cardiac tissue regions as well as the circuit of the arrhythmias, among others. In this work, we analyze the morphology of the bipolar estimated EGMs. Moreover, the operator referred to as First Temporary Derivative (FTD), which is the configuration commonly used to obtain bipolar EGM for ECGI systems, is also studied.

The FTD often introduces noise in the morphology of the obtained bipolar EGM, hence inserting apparent fragmentation. The FTD is obtained by applying the first difference to the unipolar EGM. Thus, the unipolar EGM is considered to be the voltage resulting from the exploratory electrode while the voltage of the reference electrode is obtained by shifting one sample the original unipolar EGM. This one sample delay is the cause that generates the spurious fragmentation; for this reason, this work analyzes the alternative of delaying more than one unit instead of shifting one single sample. The resulting bipolar EGM is then referred to as the delayed reference.

In order to study the time-delay effect in the bipolar-estimated EGMs, the formulation of a new DSPO $\theta_{V_{\alpha }}$ is proposed. The DSPO $\theta_{V}$ of the node under analysis is obtained by subtracting the unipolar EGM of maximum amplitude taken from its neighborhood. This unipolar EGM informs about the sense of propagation of the cardiac impulse, so the proposal of a new DSPO based on $\theta_{V}$ will result in a more realistic bipolar EGM that respects the sense of cardiac impulse propagation.

Let ${\bar{s}}_{i}$ be the node taken into consideration; the new DSPO consists of applying a time delay of α seconds to the reference node, which is the unipolar EGM of the neighborhood ${\bar{s}}_{k}$ chosen according to operator $\theta_{V}$, and of subtracting it to the unipolar EGM of node ${\bar{s}}_{i}$. Thus, Equation (12) of the new bipolar EGM at ${\bar{s}}_{i}$ can be rewritten as

(13) $$\beta \left({\bar{s}}_{i},t_{w}\right)=v\left({\bar{s}}_{i},t_{w}\right)-v\left(\theta_{V_{\alpha }}\left(C_{i}\right),\left(t_{w}-\alpha \right)\right),$$

where α corresponds to the time delay. The expression of the new DSPO $\theta_{V_{\alpha }}$ is

(14) $${\bar{s}}_{k}=\theta_{V_{\alpha }}\left(C_{i}\right),\;\;\mathrm{with}\;\;\max_{j\in \eta_{i}}\{|v\left({\bar{s}}_{j},t_{w}-\alpha \right)\left|\right\}$$

### 2.4. Data Sets

ECGI algorithms need two data sets: first, the electrocardiographic potentials over several points on the torso surface surrounding $v_{m}^{T}$ and, second, the matrix A of the heart and torso geometries [27]. To obtain potentials $v_{m}^{T}$, 250 electrodes mounted on strips are often used, which are positioned on the torso of the patient, both on the posterior and the anterior areas of the torso. These electrode strips are connected to a portable mapping system (BioSemi in Amsterdam, Netherlands). Each of these electrodes has a marker that is visible on the CT imaging. The patient undergoes thoracic noncontrast-gated CT with axial resolution of 3 mm. This CT provides us with the epicardial geometry and the positions of the torso electrodes in a single image. The torso surface potentials recorded by the 250 electrodes are sampled at 2048 Hz, and then, the registered potentials of the torso and the CT-derived geometric information are the input data for the ECGI algorithm, which constructs noninvasive epicardial potentials $v_{m}^{E}$, EGMs, isochronous maps, and repolarization patterns [6]. The ECGI methodology used in this work has been developed by Yoram Rudy Lab at Washington University in St. Louis during the last decades, and its application on human subjects is described in Reference [11]. Briefly, electrocardiographically measured potentials and CT images are obtained by the ECGI software, referred to as CADIS, which is developed in Matlab. Initially, the body surface potentials are preprocessed and image segmentation as well as and heart and torso surface meshing are achieved. After, the transfer matrix, which relates the body surface potentials to epicardial potentials, is formed using the boundary element method. The algorithm to compute epicardial potentials uses Tikhonov regularization and the generalized minimal residual iterative scheme. The regularization parameter and the number of iterations are determined using the composite residual and the conditioned L-curve, respectively. This reconstruction is performed during a single heartbeat, and it does not require the accumulation of many beats. This property makes possible an image of nonsustained and polymorphic arrhythmias as well as arrhythmias that are not hemodynamically tolerated.

Overall, these ECGI systems are being supported by a growing number of successful studies in clinical practice [7,8,9,15,28,29,30,31], but limitations in the spatial resolution still can be present in them. Other novel clinical applications are arising from advanced uses of ECGI techniques, and among them, we can find the support for the early diagnosis of arrhythmogenic right ventricular cardiomyopathy with resonance medical imaging and the help to guide the cardiac ablation of ventricular tachycardia in a completely noninvasive way [16,32,33,34,35,36].

Finally, cases chosen for this study were selected from the data sets provided by Rudy’s laboratory. The database used in this study consists of a healthy individual and a patient suffering from cardiac disease, all of them taken from previous research undertaken at Cardiac Bioelectricity and Arrhythmia Center, provided with permission by Yoram Rudy Lab. Each signal is a 1-min-long segment extracted from body surface potential mapping, and epicardial meshes were estimated from medical images. The controls were taken from existing studies in References [34,37]. The infarction patient had documented a wide infarcted tissue extension in the inferior wall [38,39,40]. We report here results with detail on the methods and restrict ourselves to one or two representative examples of the complete data sets.

## 3. Experiments and Results

Given that many of the current signal processing pipelines include one-by-one signal preprocessing and subsequent map elaboration on a given characteristic of each signal, we analyzed them separately in the available data, and for this reason, the scheme of the Experiment section is as follows. First, the information and structure of spatial-temporal autocorrelation are scrutinized both in the torso and in the epicardium signals in terms of the two preprocessing stages often used in the literature, which are baseline wander removal and the high-frequency noise filtering, and are given rationale for future development of specific preprocessing techniques overcoming the performance of current methods. Second, the spatial-temporal consistency of cardiac waveforms are analyzed and compared with the spatial consistency of maps from several relevant electrophysiological parameters obtained from said signals. Finally, simple considerations on the electrophysiological characteristics of the estimated bipolar EGMs inspired us to propose the inclusion of a time delay on DSPOs, which make estimated EGMs and catheter-recorded EGMs in practice more consistent in terms of EGM width and non-spurius fragmentation.

### 3.1. Preprocessing and Spatial-Temporal Correlations

The usual steps for analyzing cardiac signals, both in the surface ECG and in intracardiac EGM from EPS, usually start with denoising stages. Although the usual techniques are similar, settings are often different from each other. Accordingly, we aimed to scrutinize the implications of having spatial-temporal variations of the potentials in ECGI. Both in torso measured and in epicardium estimated, the common approach is what we may refer to as atomic processing, i.e., to use conventional signal processing stages for ECG and intracardiac EGM recordings, separately, for each signal in the time domain and throughout the whole spacial set of signals, irrespective of their torsal or epicardial origin.

**Figure 3:**
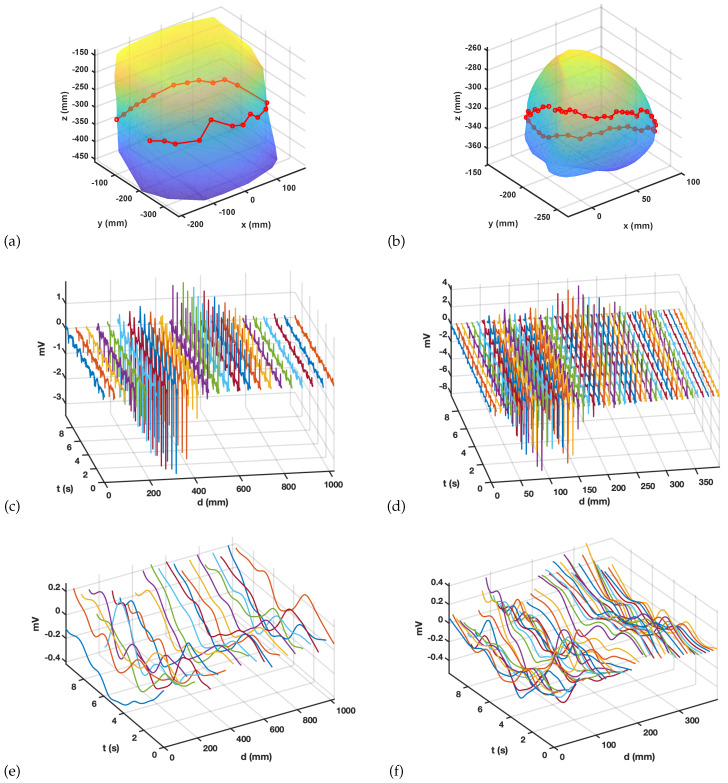
M-mode trajectories for the torso in (**a**) and epicardium in (**b**) and the corresponding measured or estimated signals in (**c**,**d**). The residuals of atomic baseline wander removal with a spline time-node spacing of 1 s are shown for both in (**e**,**f**).

For this purpose, we analyzed signals 9.7 s long, sampled at $f_{s}=2048$ Hz, for 802 points in the epicardium and for 240 points (out of 250 recorded by the system) in the torso. Note that some few torso recordings were discarded in this type of study because of their quality drop, mostly due to the loss of catheter–skin contact. Two spatial trajectories were selected, one for each spatial mesh, by selecting 23 aligned points on the torso and 45 in the epicardium, as shown in Figure 3a,b. For those trajectories, the M-mode representations allow to check for spatial-temporal similarities, as can be seen in Figure 3c,d, where the spatial dimension is measured in mm and the temporal dimension is measured in seconds. Note that, for all cases, the amplitude of the potential evolves as follows: we find the potentials being smaller at the beginning of the trajectory, then growing to a negative predominant amplitude envelope, crossing after to a positive predominant amplitude envelope, and finally, coming back to a smaller amplitude. However, even if these amplitude envelopes are grossly similar, significant differences are present between torsal and epicardial potentials, for instance, the amplitude notch of the amplitude in the predominantly negative epicardial potentials and their temporal bipolarity in the activation deflection being more present in some locations.

A baseline wander detrending was done for both sets of signals, using a cubic spline interpolator with node separation of $T_{w}$ seconds. This is a very usual procedure for denoising cardiac signals from low-frequency noise [41]. The results of atomic detrending with $T_{w}=1$ s are shown in Figure 3e,f for the same M-mode trajectory and spatial-temporal axis on the baseline wander residuals. On the one hand, the time evolution is smooth for the low-frequency trends removed by the method, which is not surprising. However, strong similarity can be observed for the time evolution shape of close spatial points. This similarity between time-changing signals in spatially close measurement locations may be referred to as spatial correlation of those measurements. It is evident that this spatial correlation is reasonably expected in the residuals of any denoising process in this kind of signals; M-mode is shown to be a very convenient tool for its characterization and for its subsequent analysis. It can be observed that spatial changes on the torso are smoother than on the epicardium, which is naturally compatible with the mix effect of the source potentials on the torso smoothing the changes on the epicardial spatial distribution of the potentials.

In order to give a more systematic description of signals and residuals, the spatial-temporal autocorrelation was obtained for each of the M-mode representations. For a spatial-temporal field x(t,s), we denote its spatial-temporal autocorrelation as $R_{x}\left(\tau ,\lambda \right)$, where τ and λ denote the usual delay in autocorrelation representations, in this case for time and space shifts, respectively. Although not the only one, a convenient way for calculation of this representation is given by $R_{x}\left(\tau ,\lambda \right)=x\left(\tau ,\lambda \right){*}_{t,d}x\left(-\tau ,-\lambda \right)$, where ${*}_{\tau ,\lambda }$ denotes the bidimiensonal convolution operator. Other estimates for the autocorrelation can be used according to data model convenience, but they are not considered here. Note that this representation discards the changes in the three-dimensional space, but in exchange, it reports the possibility of scrutinizing the joint spatial and temporal dynamics of the observed M-mode fields.

**Figure 4:**
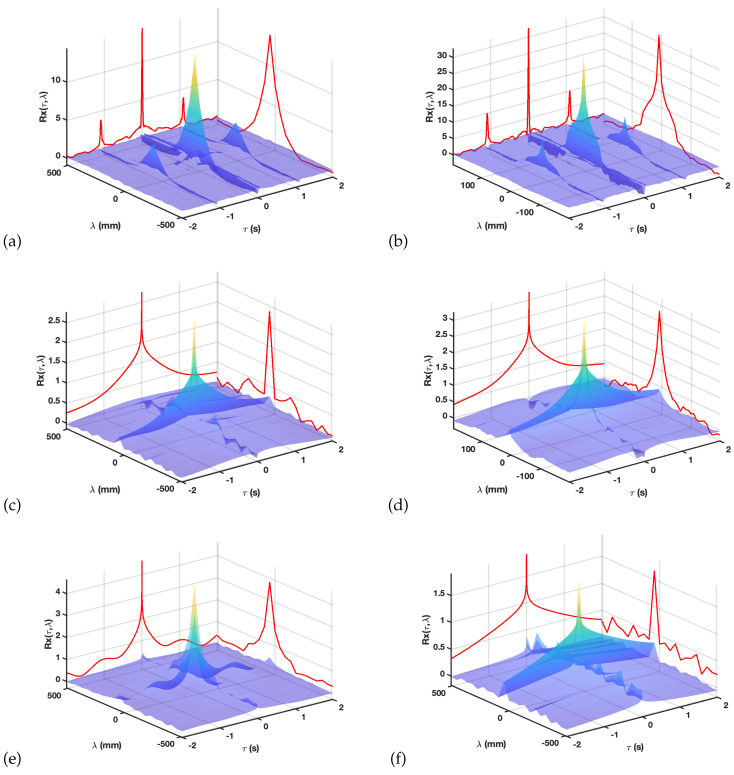
Spatial–temporal autocorrelation of the M-mode potentials (**a**,**b**) and of the M-mode baseline wander residual potentials for $T_{w}=$ 1 s (**c**,**d**) and algorithm missadaptation when using $T_{w}=$ 0.5 and 2 s (e and f, respectively) on the torso (**e**) and on the epicardium (**f**).

Figure 4a,b in the upper row show the M-mode spatial-temporal autocorrelation for the torsal and epicardial potentials. Note that the lateral walls of the 3-D plot include a representation of the purely temporal (left wall) and purely spatial (right wall), whereas their joint distribution can be studied in the surface representation. The temporal autocorrelation exhibits in both cases periodicity peaks as well as a positive behavior, mostly due to the presence of baseline wander in the measured signals, and this is more patent on the torso signals. The spatial autocorrelation shows an extremely low decay and, somehow, more complex shape in the epicardial case, which is consistent with the basic idea of the spatial correlation observed in the M-mode in the previous figure and on the EGM routinely measured with catheters during EPS study.

Figure 4c,d depict the M-mode autocorrelations for the baseline wander residuals when using $T_{w}=1$ s. We can observe positive heavy tails for the time autocorrelation in both torso and epicardium, as expected due to the low-frequency character of the residuals provided by the atomic processing built on the time signals. The spatial correlation has different profiles in torso than in epicardium residuals, though in both cases it is qualitatively similar to a close-distance main lobe and a further secondary lobe, being narrower and wider, respectively, in the torsal epicardium, which shows that the spatial properties of both domains are different. The lower row of the figure exhibits the results of two examples of baseline wander removal; Figure 4e with a too-short notch separation ($T_{w}=0.5$ s), and Figure 4f with a too-long one ($T_{w}=$ 2 s), only for the torso and for the epicardium autocorrelations, respectively. Note that the time autocorrelation of the residuals on the left exhibits the presence of periodic peaks, which usually is a sign of detrending missadaptation for a too-short time window, and that the intra-cycle trends (hence the cardiac waveforms) are being distorted. In addition, the use of a too-long window in the atomic detrending yields to a nonrealistic increase of the tails of the autocorrelation temporal residuals and to the appearance of periodic spatial peaks in the spatial residual autocorrelation.

**Figure 5:**
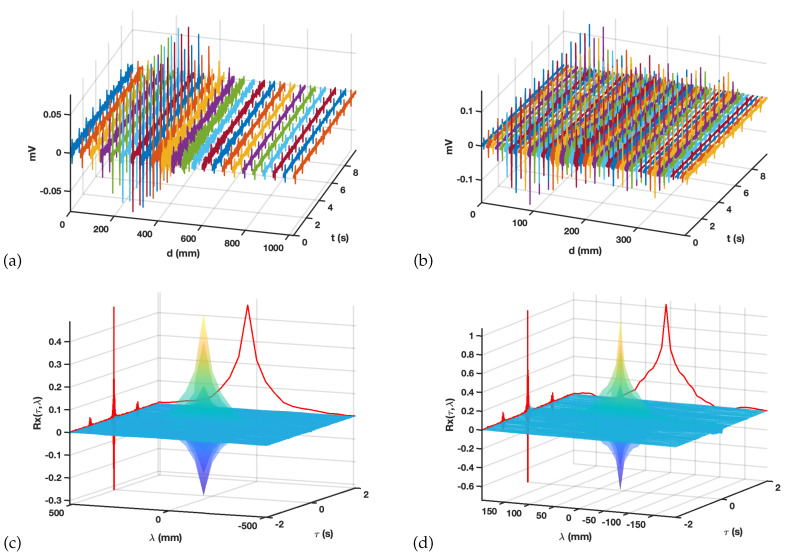
Example of high-frequency filtering of the M-mode signals: We can see the M-mode residuals in (**a**,**b**), and their spatial-temporal autocorrelations both for the torso in (**c**) and for the epicardium in (**d**).

Figure 5 shows the result of high-frequency filtering, both in the torsal and in the epicardial potentials (each signal separately), with a cutoff frequency of 100 Hz and a 9th-order zero-phase Butterworth filter. It can be observed in Figure 5a,b that the residuals resulting from this processing exhibit strong spatial and temporal autocorrelation, but again, envelope peaks are more present in the epicardial plots, which probably points to more signal distortion being done by the atomic filtering on the epicardial potentials. The temporal partial correlation shapes of the residuals shown in both sets of potentials prominent periodic peaks and the spatial partial autocorrelations are similarly broad.

Overall, the conclusions of this analysis can be summarized as follows. First, when scrutinizing the spatial-temporal autocorrelations of the M-mode potentials and of different residuals for preprocessing stages, we see that the time and space behaviors are strongly coupled, as expected. This means that both time and space should be taken into account for the preprocessing of these cardiac signals; otherwise, the results achieved could be highly suboptimal. Also, torsal and epicardial potentials have different spatial-temporal properties, so different tuning parameters should be considered in the processing stages for each of them; otherwise, strong missadaptation could be applied to the signals, hence leaving a nonsolid substrate for subsequent clinical analysis of any kind.

### 3.2. Spatial Consistency of Bipolar Potentials

This section seeks to find the most suitable operator for defining bipolar EGMs among the proposed DSPOs. For this purpose, the spatial consistency study of the previously described operators is tackled. The procedure is based on the waveform analysis according to its amplitude as well as on the coherence among potential, both in the vicinity and throughout a pathway. To begin, as the different regions of the cardiac tissue are characterized by the amplitude of the EGM, the spatial consistency according to peak-to-peak amplitudes is first visually analyzed. Afterwards, the spatial consistency is observed for a single point and their neighbors in two relevant regions of the cardiac tissue. Finally, the spatial consistency is analyzed in a dotted line that passes through different regions of the cardiac tissue. In following this procedure, those DSPO configurations showing a poor performance in comparison to what is expected in true bipolar ECG will be discarded.

**Statistical consistency of the EGM:** The statistical distributions of the EGM amplitudes obtained with each DSPO are compared in terms of their individual histograms. For each EGM at each mesh point, the amplitude was calculated as the peak-to-peak value.

**Figure 6:**
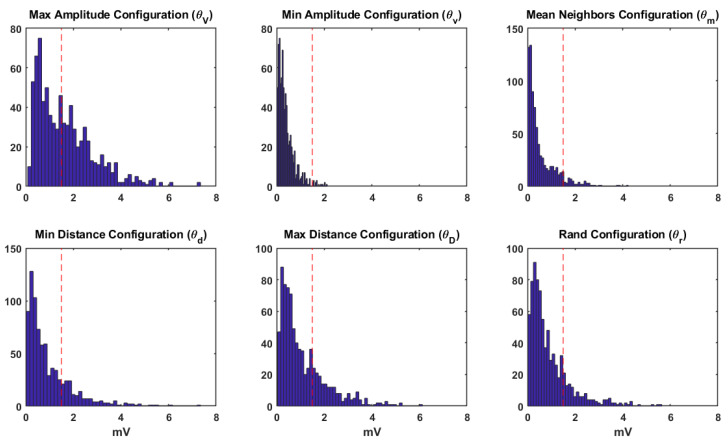
Analysis of the EGM statistical distribution in terms of the histogram of the EGM amplitudes for all the configurations.

Figure 6 shows the amplitude histograms for each DSPO. The red vertical line in the histogram represents the threshold value commonly used in the clinical practice in order to identify border regions from the healthy ones, which is 1.5 mV for ventricles [4]. The histogram for the $\theta_{V}$ operator (first graph on top left) shows 3 different amplitude zones, namely from 0 to about 1.5 mV, from 1.5 mV to about 4 mV, and larger than 4 mV. Accordingly, it could be possible that those amplitudes that are lower than the clinical threshold are well identified from scar or far field in the valves with this configuration. In the other two zones, we could identify an intermediate zone of damaged cardiac tissue, likely with slow conduction or scare edges, and a zone of normal cardiac tissue. For the case of $\theta_{v}$ and $\theta_{m}$ (remaining top graphs), most of the amplitude values are below the 1.5 mV threshold, which could lead to a wrong diagnosis, since it could be thought that all the cardiac tissues are a border region. For the cases of $\theta_{d}$, $\theta_{D}$, and $\theta_{r}$ (graphs at the bottom, from left to right), the histograms show two defined zones: The first one is between 0 and 1.5 mV, where the border region or the distant field of the valves can be identified and where most of EGM amplitudes are found. The second one is greater than 1.5 mV, where peak-to-peak amplitude values decrease exponentially, identifying this zone as healthy tissue, but this interpretation should imply that most of the locations correspond to non-healthy or far-field regions, which is highly unlikely.

**Figure 7:**
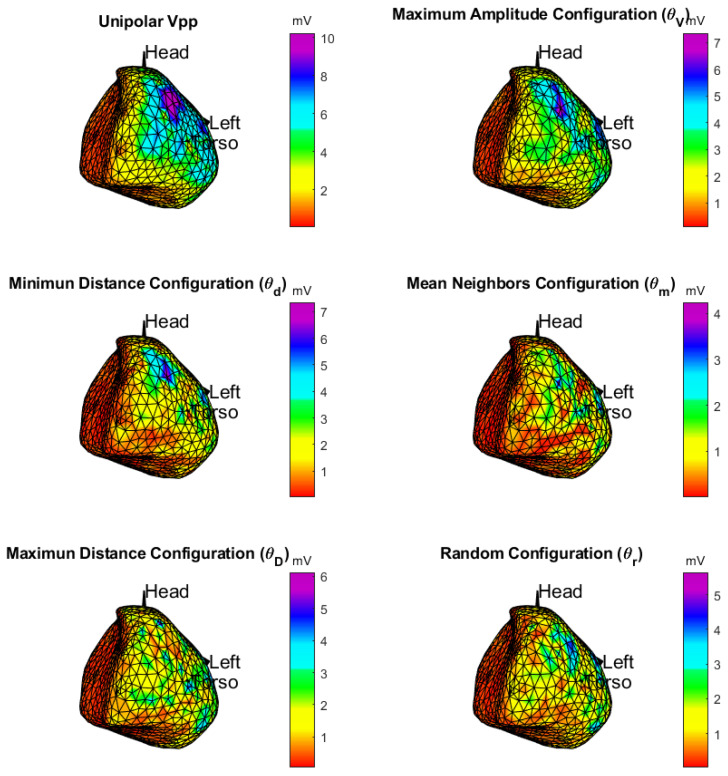
Potential maps: the top left graph is the unipolar EGM, while the remaining ones are the bipolar EGM configuration.

**Spatial consistency of the amplitude maps:**Figure 7 shows the map of peak-to-peak amplitudes of patient 7 for the unipolar EGM and each DSPO except that of the operator $\theta_{v}$, which was discarded after the previously performed analysis, where all the amplitudes were found below 1.5 mV, the value set for the border region. For each DSPO, we can see that the different regions are consistent with the patient’s physiology- and pathology-known substrate, namely the right side, the zone of the valves, and the inferior part of the ventricle. The color scale of each map is adjusted to the maximum peak-to-peak amplitude of each DSPO. In the unipolar EGM map, the scar region is located in the inferior left wall of the ventricles and the region of the valves also corresponds to the red color assigned to smaller amplitudes. In the lateral-upper wall of the ventricles, we can see a zone of high amplitude that degrades toward the apex and towards the inferior wall of the ventricles, where the scar is located. Operator $\theta_{V}$ exhibits spatial consistency with respect to the unipolar EGM, which allows us to determine different regions in the cardiac tissue, and the scar region can be identified in the inferior left wall as well as the valve region. Similarity in the spatial consistency is lower with $\theta_{d}$ configuration, with more regions of low amplitudes which seem to be confused with the scar region. In the configuration with operators $\theta_{m}$, $\theta_{D}$, and $\theta_{r}$, there is a markedly reduced spatial consistency with respect to the unipolar EGM, since very small amplitudes in the former appear in regions of higher amplitudes in the latter, making the scar region very ambiguous. In general, correlation coefficients between maps were at most moderately high, as they measure different properties. For instance, the correlation coefficient of DSPO $\theta_{V}$ obtained of the peak-to-peak voltage in bipolar vs unipolar EGMs was 0.7140, whereas for $\theta_{d}$, it was 0.7804.

After the spatial consistency analysis of the amplitude and the statistical study of the previous section, we can conclude that the two $\theta_{V}$ and $\theta_{d}$ DSPOs exhibit a behavior more similar to that of an actual bipolar EGM. Note that this result is in compliance with what really happens in EPS as far as the amplitude is greater whenever the dipole is in parallel with the propagation front. Also, the closer the electrodes, the greater the amplitude, because the far-field influence is lower in this case.

For the above reasons, the rest of the analysis of the present study is carried out only with these two configurations.

**Spatial consistency on neighbors:** Now, we analyze the spatial consistency with respect to the angle and distance at a point and its closest neighbors in two regions of cardiac tissue, namely scar and healthy myocardium tissue. For this purpose, we use the M-mode representations of the EGMs, which are defined as the time–space representation signal measurement in a set of points, i.e., $\mu (d_{j},t)$ or $\mu (\phi_{j},t)$, where $j\in \eta_{i}$, *d* is the distance and ϕ is the angle.

**Figure 8:**
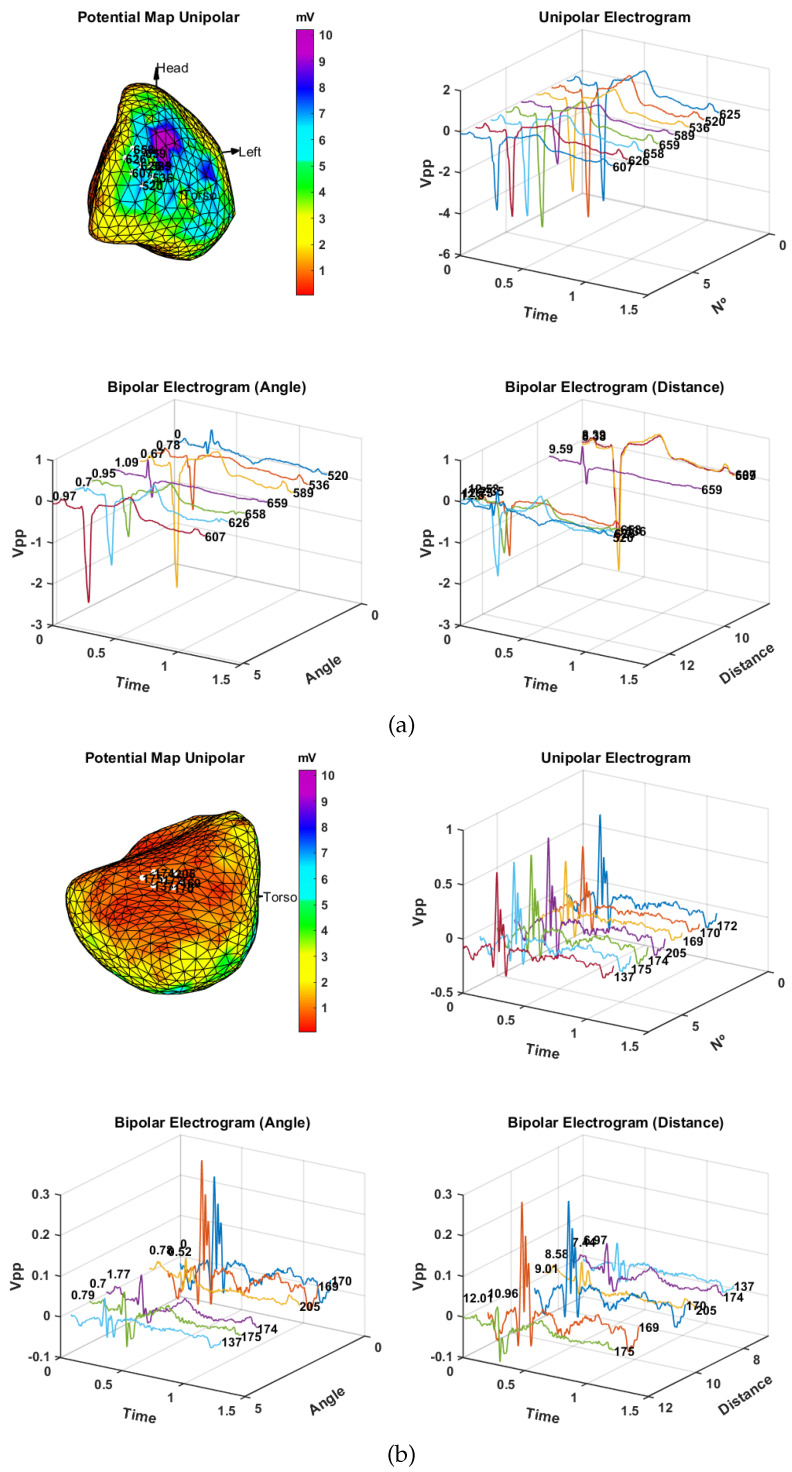
Closest neighbor consistency in a healthy region (**a**) and on a scar region (**b**) of the infarcted patient: On each panel is (up, left) potential map, (up, right) M-mode of unipolar EGMs, and (down) spatial consistency of bipolar EGM with respect to the angle (left) and distance (right). Distance between nodes is in mm, time is in s, angle is in radians, and $V_{pp}$ is in mV. The selected locations are highlighted in the maps with white points and their reference number.

Figure 8a shows the analysis of the closest neighbors of a point in the healthy region of the cardiac tissue. In the potential map panel at the upper left graph, one can see the location of the neighborhood of points; the remaining graphs show the unipolar and bipolar EGMs of each of these neighbors ordered according to the angle and distance with respect to the central point of the neighborhood. In this case, point 589 corresponds to that of the minimum distance and maximum amplitude in the closest neighborhood of points. In the bottom row of the figure, we can see that the bipolar EGM obtained with point 589 clearly corresponds to the EGM of greater amplitude among the neighbors. In addition, the direction of propagation that has the impulse is better compensated in the bipolar EGM of greater amplitude, since bipolar EGMs in opposite angles and in the direction perpendicular to propagation have reduced amplitude.

In Figure 8b, we show the analysis of the nearest neighbors of a point in the epicardial scar region. In the map of potentials of the upper panel, we can see the location of the neighborhood of points of the scar zone. In this case, the scar region is located in the inferior left ventricular wall of the heart, which agrees with the patient clinical history. If we analyze the unipolar EGMs of the neighborhood, we can see that all of them are fragmented and that they have amplitudes less than 0.7 mV. In EPS, the amplitudes of unipolar EGM between 3 mV and 5 mV are considered the border region and amplitudes less than 3 mV are considered the scar region [42]. This can be a problem, since during the electrophysiology analysis, it may be thought that the measure is wrong or that we are outside the cardiac tissue; in addition, these signals are so small that it is impossible to determine whether the ECG is fragmented. On the other hand, if we observe the bipolar EGMs of greater amplitude, they consistently hold the EPS criteria of 0.5 mV used in clinical practice for the scare region.

**Spatial consistency on lines:** An additional analysis of M-modes was performed in order to study the two DSPOs operators found to be optimal, $\theta_{V}$ and $\theta_{d}$. As referred before, M-modes are suitable representations to analyze the spatial consistency in a line of mesh points, passing through different regions of the cardiac tissue. Bipolar EGM of each selected consecutive point of the line are represented according to the DSPO selected. The purpose is to see the amplitude changes on the unipolar and bipolar EGMs between the cardiac tissue region so that we can choose the more faithful DSPO for the representation of the bipolar EGM signal in electrophysiology.

**Figure 9:**
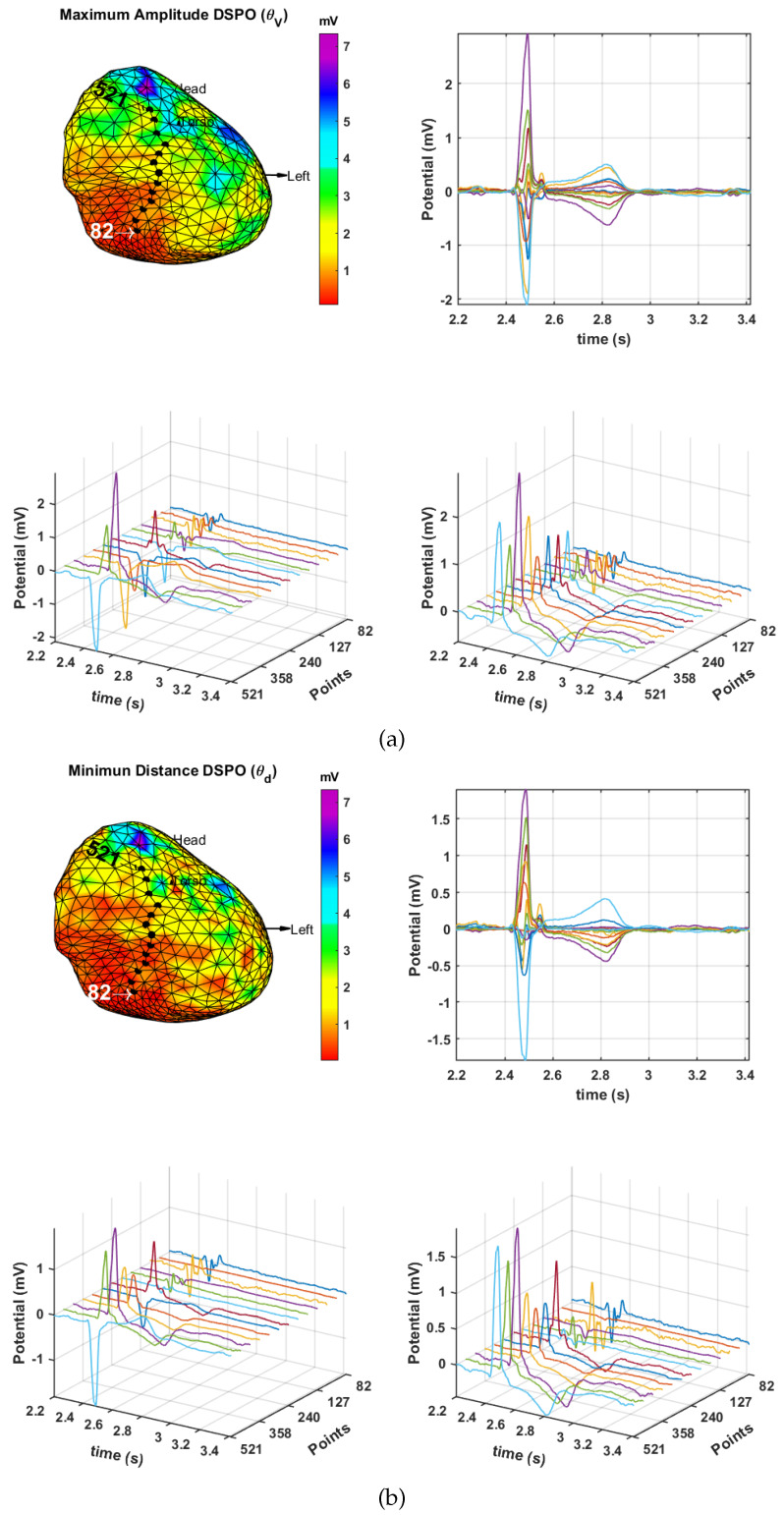
Line of points starting from epicardial scar up to a healthy region of cardiac tissue in the infarction patient with bipolar EGMs using the $\theta_{V}$ (**a**) and the $\theta_{d}$ (**b**) criteria: (up, left) map of potentials, (up, right) bipolar EGMs of the line of points, (down, left) M-mode of the bipolar EGMs of the dotted line, and (down, right) M-mode of bipolar EGMs adjusted by using the correlation coefficient.

The upper panel of Figure 9a shows the potential map using the DSPO $\theta_{V}$ (on the left) and the bipolar EGMs (on the right) of the dotted line that goes from the scar region (point 82) up to the healthy region (point 521). The maximum amplitude attained is approximately 3 mV, corresponding to a bipolar EGM of the healthy region of the cardiac tissue, which results in the greatest amplitude deflection. In addition, the deflections of bipolar EGMs that represent the depolarization and repolarization of cardiac tissue cells are clearly represented. On the other hand, the M-mode of the bipolar EGMs of the dotted line is shown on the lower panel. Thus, the spatial consistency of the bipolar EGM, obtained with the DSPO $\theta_{V}$, can be analyzed in the lower left graph. The amplitude of the first bipolar EGM, corresponding to the scar region, is less than 0.5 mV; in the border region, the amplitudes increase (between 0.5 mV and 1.5 mV); and finally, the last bipolar EGM, in the healthy region, attains an amplitude greater than 1.5 mV. This spatial consistency is better observed in the lower right graph, where the M-mode is ordered in positive direction. This is carried out with the correlation coefficient applied to the bipolar EGMs of the dotted line, which gives the polarity of the bipolar EGM; when negative, it is multiplied by −1 to change its direction.

Similarly, Figure 9b shows the result in the same line of points but now performed with the $\theta_{d}$ DSPO operator. With respect to the spatial consistency of the M-modes, we can see that the bipolar EGMs of lower amplitude (less than 1.5 mV) are located in the scar region and that, as long as the healthy region is approached, the amplitude grows. The maximum amplitude of the bipolar EGM attained in a healthy region takes on approximately in this case the value of 2 mV. The deflections of the bipolar EGMs are clearly identified as well.

According to this analysis, we can assert that the information provided by both configurations is similar, though the $\theta_{V}$ operator elicits a signal with better identification signs: greater amplitude and deflections of the bipolar EGM clearly represented.

### 3.3. Time Delay from Empirical Considerations

In this section, we analyze the morphology of the FTD configuration and of the bipolar EGMs obtained with the DSPO $\theta_{V}$ configuration, which, according to a previous study, is the one that better met the electrophysiological criteria. The morphology of $\theta_{V}$ and FTD presents some ambiguities with respect to the analyzed literature [43,44], which is why a more realistic approximation of the obtained bipolar EGMs is subsequently studied.

**Figure 10:**
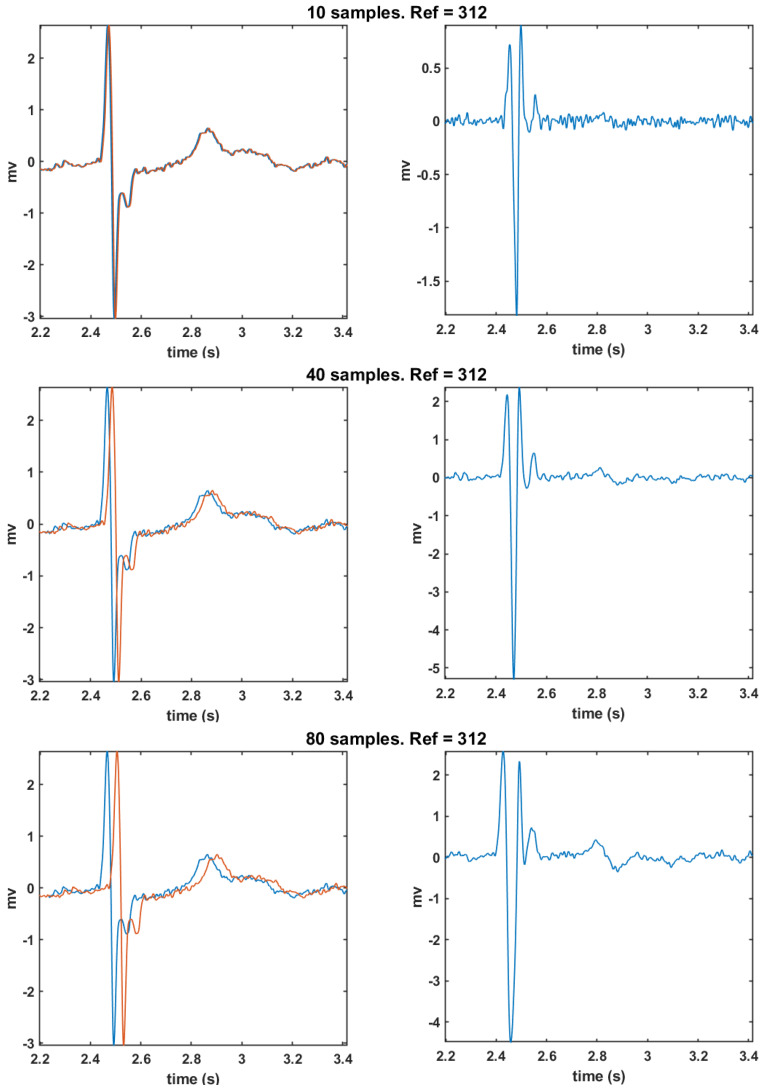
Bipolar EGMs obtained with the delayed reference operator: Graphs on the left column depict the unipolar EGM (point 312), in red, and the same unipolar EGM delayed, in blue. Graphs on the right column show the bipolar EGM obtained as the subtraction of the unipolar ones taken from the left. From top to bottom, the results correspond to delays of 10, 40, and 80 samples.

**Morphology analysis for FTD:**Figure 10 shows the bipolar EGM of mesh point 312, which corresponds to a healthy cardiac tissue. It can be seen that the magnitude of the time shift applied to the unipolar EGM affects the waveform of the resulting bipolar EGM. With a 10-sample shift, the morphology of the resulting bipolar EGM presents fragmentation in the depolarization wave and is accompanied by noise. In the case of a shift of 40 samples, the bipolar EGM does not exhibit fragmentation in the depolarization wave and the noise is smaller than in the previous case. For a time shift of 80 samples, the bipolar EGM shows non-fragmentation in the depolarization wave, though it introduces greater and nonphysiological width in its depolarization.

According to this analysis, we can see that modifying the time delay to more than one sample in the FTD operator generates better bipolar ECG shapes. Specifically, a time shift of 40 samples applied to the reference unipolar EGM provides a bipolar EGM similar to those usually visualized in EPS. Therefore, in the view that the introduction of a time shift parameter outperforms the waveform morphology, and that, among the proposed DSPO, the best bipolar EGM is obtained with $\theta_{V}$, we will conduct a study by means of $\theta_{V_{\alpha }}$

**Figure 11:**
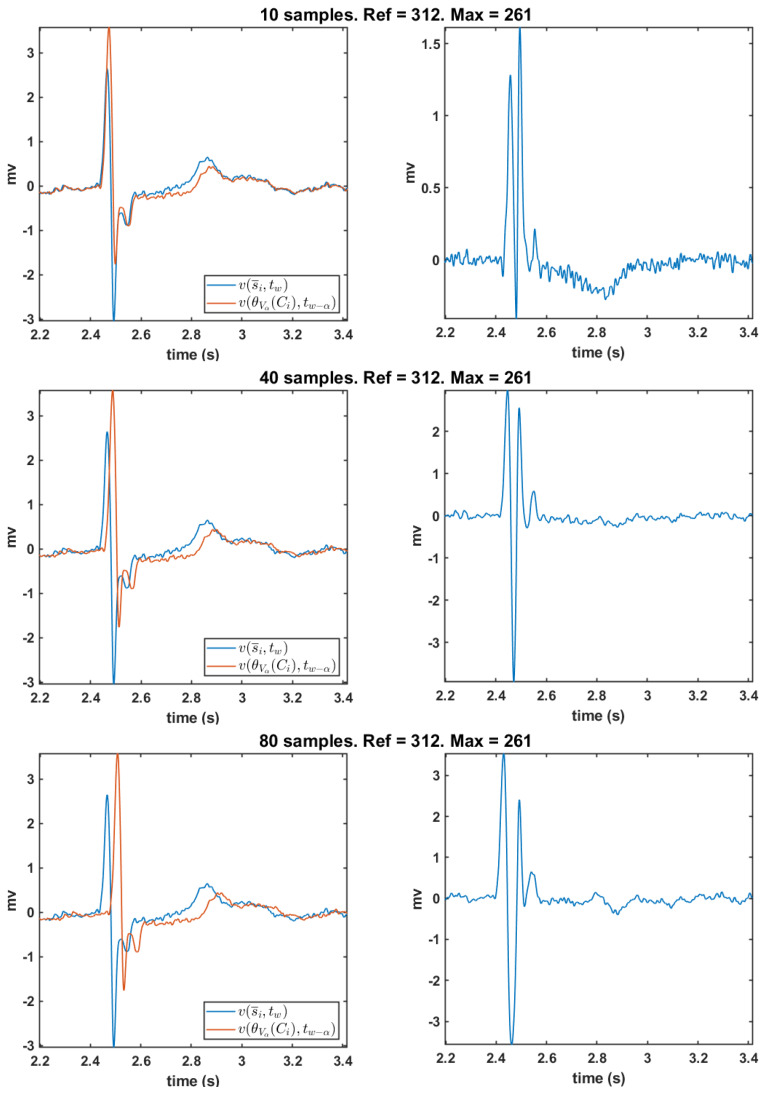
Left column depicts, in blue, the unipolar EGM of the node under study, and, in red, the delayed unipolar EGM of the neighborhood chosen according to DSPO $\theta_{V_{\alpha }}$. Right column exhibits the resulting bipolar EGMs obtained by subtraction. Panels, from top to bottom, represent the computation of bipolar EGMs for different time delays $\left(\alpha =\frac{\#\;\mathrm{samples}}{f_{s}}\right)$, namely 10, 40, and 80 time shift samples, respectively.

**Morphology analysis for $\theta_{V}$ and possible configuration:**Figure 11 shows the resulting bipolar EGM using the new DSPO $\theta_{V_{\alpha }}$ for several delay values of α seconds. As in the previous case, the bipolar EGM is determined at mesh point 312. Graphs on the left column depict the unipolar EGM, in blue, of the mesh point under analysis and the shifted unipolar EGMs $\theta_{V_{\alpha }}$, in red. The resulting bipolar EGMs for each time shift are represented on the left column. From top to bottom, the delays are considered to be 10, 40, and 80 samples. For a discrete time shift of 10 samples ($\alpha =10/f_{s}$ seconds, where $f_{s}=2048$ Hz is the sampling frequency), the bipolar EGM presents fragmentation in the depolarization wave (top right) as well as noise. Additionally, the amplitude with respect to the location of the reference point in the cardiac tissue is low. The remaining bipolar EGMs (middle and bottom left graphs) do not exhibit fragmentation anymore in the depolarization wave and they report a reasonable amplitude by considering that the cardiac tissue under consideration is corresponding to a healthy region. Finally, notice that the depolarization duration of the 80-sample shift bipolar EGM (bottom left graph) is greater.

In summary, we have seen that, working with the delayed reference operator, the time shift of 40 samples is the best option for a bipolar EGM morphology and the closest one to the usually observed in EPSs. Moreover, in the example case of point 312 of the mesh, corresponding to a healthy region of cardiac tissue, both cases, the shifted difference and $\theta_{V_{\alpha }}$, exhibit similar waveform morphologies. Now, the behavior of these operator to obtain bipolar EGMs is checked in a point of the border region of the mesh.

**Analysis of $\theta_{V_{\alpha }}$ in border region and fragmentation:** This section tackles a comparative study of operators $\theta_{V}$, $\theta_{V_{\alpha }}$, and delayed reference at point 124 of the mesh, which corresponds to a border region of the cardiac tissue; the analysis aims to scrutinize morphology and amplitude. Wherever needed, the delay is set to be 40 samples $\left(\alpha =\frac{40}{f_{s}}\right)$. Just for reminder purposes, the peak amplitudes which define each region of the cardiac tissue in bipolar EGMs are <0.5 mV scar region, between 0.5 mV and 1.5 mV border region, and >1.5 mV healthy region. After the comparison study, a fragmentation analysis of the bipolar EGMs is addressed for the DSPO $\theta_{V_{\alpha }}$.

**Figure 12:**
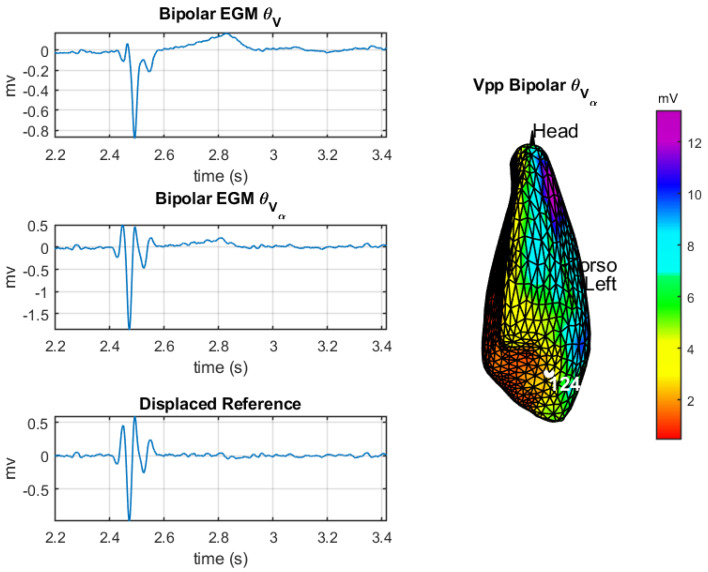
DSPO comparison: the left column depicts the obtained bipolar EGM of point 124 of cardiac tissue. From top to bottom are $\theta_{V}$, $\theta_{V_{\alpha }}$, and *delayed reference*. The right-hand plot represents the amplitude map of the DSPO $\theta_{V_{\alpha }}$ and the location of the analyzed point.

The left column of Figure 12 shows the bipolar EGM obtained with the DSPOs $\theta_{V}$, $\theta_{V_{\alpha }}$, and delayed reference of point 124 of the mesh, while the right column depicts the amplitude map of DSPO $\theta_{V_{\alpha }}$ and the location of point 124 in a border region. Regarding the morphology of the bipolar EGMs, the waveforms obtained with $\theta_{V_{\alpha }}$ (curve in middle graph) and delayed reference (curve in bottom graph) are similar but different to $\theta_{V}$ (curve in top graph). Regarding amplitudes, the range of values for both $\theta_{V_{\alpha }}$ and delayed reference is different: between −1.5 and 0.5 mV for the former and between −0.6 and 0.5 mV for the latter. The analysis of amplitudes reveals that the values obtained with $\theta_{V_{\alpha }}$ comply with the peak amplitudes of a border region. Thus, we can see that the proposed DSPO $\theta_{V_{\alpha }}$ is a reasonable option to obtain the bipolar EGM in the ECGI systems as far as the amplitude limits for the bipolar EGM visualized in the EPSs are respected.

The study of the fragmentation of the bipolar EGM obtained with the DSPO $\theta_{V_{\alpha }}$ is addressed in three different regions of the cardiac tissue. The criteria used to detect fragmentation are based on the number of notches present in the deflection of the bipolar EGMs, and its severity was manually assessed by the authors for all bipolar EGMs, following the electrophysiological criteria of Reference [45] and closely supervised by an electrophysiologist.

**Figure 13:**
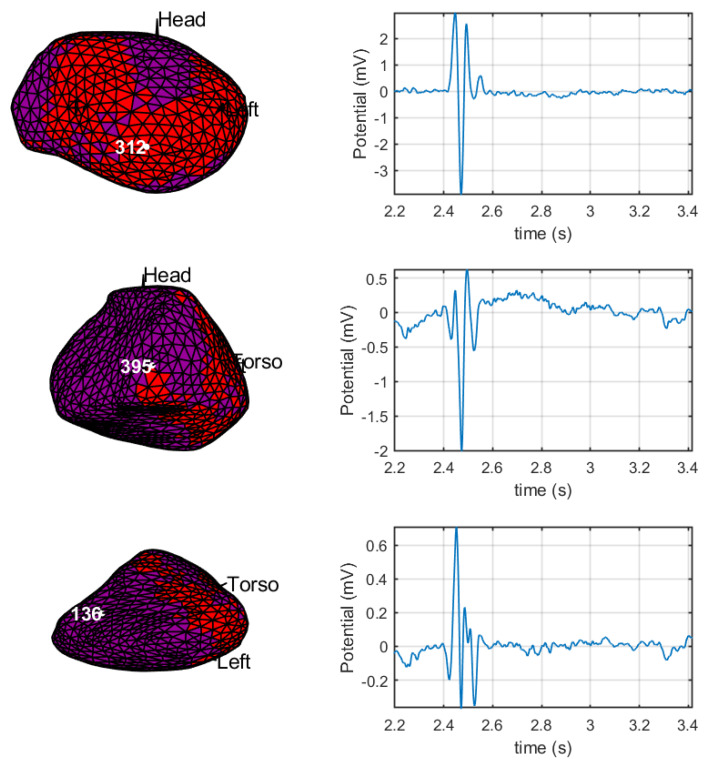
Fragmentation maps and bipolar EGMs with the DSPO $\theta_{V_{\alpha }}$: The left column shows the fragmentation maps and the selected points of the mesh. From top to bottom are 312, 395, and 136. The curves on the right column depict the bipolar EGM for each corresponding points of the left.

Figure 13 shows the selected points to be studied in the fragmentation maps in the left column and the corresponding bipolar EGMs obtained with the $\theta_{V_{\alpha }}$ operator in the right column. Point 312 of the mesh (upper row), located in a healthy region, exhibits a non-fragmented bipolar EGM. Point 395 (middle row), located in a border region of the cardiac tissue, also exhibits a non-fragmented bipolar EGM; nevertheless, in the fragmentation map, we can observe that the bipolar EGMs in the nearby region are fragmented. Finally, point 136 (lower row), which is inside the scar region, elicits a fragmented bipolar EGM with an amplitude corresponding to this region.

In general, we can see that $\theta_{V_{\alpha }}$ is the best option among the proposed DSPOs to obtain the bipolar EGM in the ECGI systems. The reason it due to the waveforms provided by the different operators, which are visually compared to those observed during EPS; from this study, $\theta_{V_{\alpha }}$ is the operator which better approached actual bipolar EGMs, offering the most realistic morphology. Furthermore, since unipolar EGM of maximum amplitude is used, we are respecting the propagation direction of the cardiac impulse, obtaining bipolar EGMs in the border regions of cardiac tissue that in turn respect the limits of amplitude studied in EPS. In addition, with respect to morphology and fragmentation, we have seen that reasonably acceptable results are obtained.

## 4. Discussion and Conclusions

The objective of the ECGI is to obtain a detailed description of the spatial-temporal pattern of the cardiac electrical activity in a noninvasive way [46]. Traditional noninvasive ECG techniques have limited ability to determine the location of electrical events in the heart with acceptable resolution. On the other hand, epicardial potentials reflect details of cardiac electrical activity with high resolution [6,7]. In several studies, the capacity to calibrate the distributions of epicardial potentials and isochrones from measurements of surface potentials has been demonstrated [5,47,48,49,50].

The local activation time is used in the generation of electroanatomical activation maps and the interpretation of the circuits that maintain some arrhythmias. The best criterion to obtain the activation time is given by unipolar EGMs, and it corresponds to the time instant of maximum descending slope of the unipolar EGMs, either filtered or not. In the case of bipolar EGM, a criterion for local activation is less known, but the point of maximum amplitude of the first peak of the EGM that coincides with the maximum slope of the unipolar EGM is often used in practice [25]. Another relevant characteristic of the EGMs is their voltage. The amplitude of a bipolar EGM is related to the intensity and amount of activation of the underlying cardiac tissue. However, both the interelectrode distance and the activation direction are decisive in this regard [4]. As mentioned above, there are invasive procedures in the EPS for the identification of arrhythmias. Currently, these studies are aided by different cardiac mapping techniques and navigation systems that make EPSs much more efficient [51,52,53]. Within these invasive procedures, we have the alternatives of contact and noncontact recordings and their use to create electroanatomical maps. Although both contact and noncontact electroanatomical mapping can be used to facilitate ablation of arrhythmias, the noncontact mapping has the potential advantage to be applicable in cases where the arrhythmia cannot be tolerated during the EPS [54,55,56,57].

In this work, we have applied conventional signal processing to every cardiac signal in both torsal and epicardial surfaces to find relations among the potential signals as well as those resulting from preprocessing stages. This type of unidimensional analysis is referred to here as atomic study. Regarding the potentials in one single surface, e.g., the measured ones at torso, or the estimated ones at epicardium, the spatial correlation among close signals is visually patent. On the other hand, when comparing signals in equivalent positions of different surfaces, the overall behaviour is similar in both cases, though significant differences are found in terms of their amplitude and temporal properties. The study also covers the signal morphology resulting from preprocessing stages; this work addresses baseline wander and high-frequency noise through spatial–temporal visualization of their residuals, resulting in the same conclusions, namely spatial correlation among close signals in one specific surface but differences among different surfaces. These visual experiments are mathematically supported by means of a two-dimensional study of autocorrelation in both time and space dimensions. The first conclusion that arises is that the development of specific preprocessing would be welcome for ECGI applications by exploiting the existing spatial correlation in order to outperform the results attained by conventional atomic processing. Second and based on the different spatial-temporal properties observed in torsal and epicardial surfaces, different tuning parameters should be considered for each of them. Taking this into consideration, the development of novel signal preprocessing methods for ECGI analysis may allow to outperform the possible suboptimal results that are likely obtained currently in subsequent clinical analysis with ECGI applications.

Regarding the type of potentials, both unipolar and bipolar EGMs have significant interest in EPS; nonetheless, ECGI only reports information regarding unipolar EGMs. However, when diagnosis relies on the EGM morphology and time characteristics, clinical procedures are defined in terms of bipolar EGMs. This work is aimed at covering this type of clinical analysis, so one of our contributions is the construction of bipolar EGMs from the set of available unipolar ones retrieved at the epicardial surface after solving the inverse problem. The proposed model to obtain bipolar EGMs is developed as follows: a point in the epicardial surface mesh can be considered to simulate the exploratory electrode of a catheter, whereas the reference electrode is taken to be one of the points in its closest neighborhood; this reference electrode is chosen according to the criteria defined by one digital signal processing operator (DSPO). The bipolar EGM is finally obtained by subtraction. Several configurations of the DSPO have been tested, and a spatial study of consistency has been addressed relying on clinical criteria to determine which of them is the best suited. Among all the studied configuration, the DSPO with better properties is $\theta_{V}$, which chooses the neighbor with maximum amplitude as the reference node. This conclusion is achieved because the amplitudes exhibited by the resulting bipolar potential are in compliance with the clinical guidelines followed in EPS for the different surface regions, namely scar, border, and healthy tissue. One drawback of this operator is that it introduces severe noise which may be misinterpreted as fragmentation in the depolarization section of the EGM. For this reason, we were inspired in the first temporary derivative, which is commonly used in clinical practice, to apply a small delay to the reference bipolar EGM resulting from the operator $\theta_{V}$. This new operator is referred to as $\theta_{V_{\alpha }}$ and is the one which, in addition to preserving the clinical properties, reports the most realistic waveform morphology. Let us note that, in fact, this operator offers the better choice when the discrete time shift is set to be 40 samples, which, for a sampling frequency of 2048 Hz, corresponds to a delay of α=0.0195 s. Taking into account that the conduction speed of the cardiac tissue oscillates between 0.3 to 0.5 m/s, the impulse will have run approximately 6 mm (0.3×0.0195=0.0059 m) during the time α, which is a distance equivalent to the interspace size between electrodes in a conventional catheter.

Several limitations are evident in our study. Our processing methods have been described and their implementation is simple, so that they can be replicated in other data sets by any group working in the field. We would like to stress that one of our main purposes in this and in the companion work [22] was to call attention to the convenience of giving increasing consideration to the spatial-temporal nature of the problem when processing ECGI signals. We did not have available either MRI data or simultaneously recorded bipolar EGMs, which should be taken into special consideration when designing future and specific studies. A quality analysis of fragmented EGMs in ECGI is surely a topic to be also scrutinized with detail, taking into account spatial-temporal considerations, whereas it deserves specific dedication and it would be beyond the scope of the present work but within the horizon of future work. We should also note that the use of M-mode plots was intended to give a better view of spatial-temporal properties in the present study and in other studies by our group. Whereas it is complicated to present a single best orientation, our team spent our best efforts and time to select the views that are currently included in this set of figures, though we are aware that some plots turned dense in any case.

The results of the experiments in the present work encourage the simultaneous consideration of time and space domains in future digital processing subsystems in the ECG field from a two–fold contribution, namely from preprocessing considerations on the spatial-temporal structure of the autocorrelation function of biopotentials and from the impact of the bipolar configuration on their properties. In the companion paper, the scope of the spatial-temporal analysis is scrutinized from its impact on cardiac indices when they are measured from ECGI-based biopotentials.

## Figures and Tables

**Table 1:** Digital Signal Processing Operator (DSPO) definitions to choose the neighbor node ${\bar{s}}_{k}$ which characterizes the reference bipolar EGM associated to a given point ${\bar{s}}_{i}$ in the Electrocardiographic Imaging (ECGI) mesh.

DSPO	${\bar{s}}_{k}$	$v({\bar{s}}_{k},t_{w})$
Amplitude	$\theta_{V}\left(C_{i}\right)$	$\max_{j\in \eta_{i}}\left\{|v({\bar{s}}_{j},t_{w})|\right\}$
	$\theta_{v}\left(C_{i}\right)$	$\min_{j\in \eta_{i}}\left\{|v({\bar{s}}_{j},t_{w})|\right\}$
Random	$\theta_{r}\left(C_{i}\right)$	${\mathrm{rand}}_{j\in \eta_{i}}\left\{v({\bar{s}}_{j},t_{w})\right\}$
Mean	$\theta_{m}\left(C_{i}\right)$	${\mathrm{mean}}_{j\in \eta_{i}}\left\{|v({\bar{s}}_{j},t_{w})|\right\}$
Distance	$\theta_{D}\left(C_{i}\right)$	$\max_{j\in \eta_{i}}\left\{|{\bar{s}}_{i}-{\bar{s}}_{j}|\right\}$
	$\theta_{d}\left(C_{i}\right)$	$\min_{j\in \eta_{i}}\left\{|{\bar{s}}_{i}-{\bar{s}}_{j}|\right\}$
